# Loss of Apelin Augments Angiotensin II-Induced Cardiac Dysfunction and Pathological Remodeling

**DOI:** 10.3390/ijms20020239

**Published:** 2019-01-09

**Authors:** Teruki Sato, Ayumi Kadowaki, Takashi Suzuki, Hiroshi Ito, Hiroyuki Watanabe, Yumiko Imai, Keiji Kuba

**Affiliations:** 1 Department of Biochemistry and Metabolic Science, Akita University Graduate School of Medicine, 1-1-1 Hondo, Akita 010-8543, Japan; satot@med.akita-u.ac.jp (T.S.); kadowakiayu@med.akita-u.ac.jp (A.K.); tksuzuki@iwate-med.ac.jp (T.S.); 2 Department of Cardiology, Akita University Graduate School of Medicine, Akita 010-8543, Japan; hitomed2@gipc.akita-u.ac.jp (H.I.); hirow@doc.med.akita-u.ac.jp (H.W.); 3 Laboratory of Regulation of Intractable Infectious Diseases, National Institute of Biomedical Innovation, Health and Nutrition, Ibaraki, Osaka 567-0085, Japan; y-imai@nibiohn.go.jp

**Keywords:** apelin, APJ receptor, angiotensin II, angiotensin-converting enzyme 2, ACE inhibitor, transforming growth-factor beta, heart failure

## Abstract

Apelin is an inotropic and cardioprotective peptide that exhibits beneficial effects through activation of the APJ receptor in the pathology of cardiovascular diseases. Apelin induces the expression of angiotensin-converting enzyme 2 (ACE2) in failing hearts, thereby improving heart function in an angiotensin 1–7-dependent manner. Whether apelin antagonizes the over-activation of the renin–angiotensin system in the heart remains elusive. In this study we show that the detrimental effects of angiotensin II (Ang II) were exacerbated in the hearts of aged apelin-gene-deficient mice. Ang II-mediated cardiac dysfunction and hypertrophy were augmented in apelin knockout mice. The loss of apelin increased the ratio of angiotensin-converting enzyme (ACE) to ACE2 expression in the Ang II-stressed hearts, and Ang II-induced cardiac fibrosis was markedly enhanced in apelin knockout mice. mRNA expression of pro-fibrotic genes, such as transforming growth-factor beta (TGF-β) signaling, were significantly upregulated in apelin knockout hearts. Consistently, treatment with the ACE-inhibitor Captopril decreased cardiac contractility in apelin knockout mice. In vitro, apelin ameliorated Ang II-induced TGF-β expression in primary cardiomyocytes, accompanied with reduced hypertrophy. These results provide direct evidence that endogenous apelin plays a crucial role in suppressing Ang II-induced cardiac dysfunction and pathological remodeling.

## 1. Introduction

Apelin is an endogenous peptide ligand for dreceptor (or Aplnr) and has potent positive inotropic activity and vasodilatory action [1,2,3]. APJ is a G protein-coupled receptor that shares significant homology with the angiotensin II type 1 receptor (AT1R) [4]. The apelin–APJ axis regulates cardiovascular functions including blood pressure, cardiac contractility, and fluid balance, and thereby exerts beneficial effects on cardiovascular systems [5,6]. Treatment with apelin peptide in vivo improves the cardiac contractility and output in myocardial infarcted rats and normal mice, and protects the heart from pressure overload, isoproterenol-induced injury, or ischemia reperfusion injury [7,8,9]. In studies of apelin knockout and APJ knockout mice, we and others have demonstrated that the endogenous apelin–APJ axis regulates the heart contractility associated with aging, exercise, and pressure overload; in the absence of apelin or APJ expression, these mutant mice show contractile dysfunctions [8,10,11]. By contrast, APJ was identified as a dual receptor to mediate cardiac hypertrophy [12,13] via β-arrestin signaling [14,15]. Thus, the precise role of endogenous apelin signaling in heart function remains elusive.

Cardiac hypertrophy is an adaptive response to maintain cardiac function in the event of an increased workload, although prolonged cardiac hypertrophy can lead to heart failure via the ischemia of cardiomyocytes [16,17]. In addition, cardiac fibrosis adversely increases tissue stiffness and impairs ventricular function. Consistently, cardiac hypertrophy and the development of fibrosis correlate with the deterioration of myocardial function in heart failure [18,19,20]. Thus, cardiac hypertrophy and fibrosis are key features of heart failure, and apelin’s inotropic and protective roles in the heart are attractive as a potential therapeutic candidate for treating heart failure.

Apelin has also been identified as a substrate for angiotensin-converting enzyme 2 (ACE2), which cleaves apelin with similar efficiency as its primary substrate angiotensin II (Ang II) [21,22,23]. ACE2 is a negative regulator of the renin–angiotensin system (RAS) that inactivates Ang II by converting it to angiotensin 1–7 (Ang 1–7) [21,22,23], whereas angiotensin-converting enzyme 1 (or ACE1) activates RAS by converting Ang I to Ang II. ACE2 was also identified as a regulator of heart failure [24,25,26], diabetic nephropathy [27,28], acute lung failure [29,30], and the SARS (severe acute respiratory syndrome) coronavirus receptor [31,32].

Apelin-APJ signaling has been shown to counteract the effects of Ang II on AT1R in defined physiological and pathophysiological settings. For instance, in hypertensive rat models, the continuous infusion of Ang II downregulates apelin and APJ expression [33]. We have shown that Apelin upregulates ACE2 expression. Apelin-induced ACE2 downregulates Ang II signaling while enhancing Ang 1–7–Mas receptor signaling in aged or failing hearts [34]. On the other hand, it has been suggested that the counter-balancing effects of the apelin–APJ axis on Ang II–AT1R signaling are mediated by receptor dimerization. The APJ receptor physically interacts with AT1 receptors, and exogenous apelin negatively regulates Ang II–AT1R signaling in in vitro cell culture systems and in an in vivo atherosclerosis model [35]. However, whether endogenous apelin counteracts the over-activation of RAS in the heart remains unexplored.

In this study, we aimed to determine the effects of endogenous apelin on Ang II-induced pathology in the heart. We showed that Ang II-mediated cardiac dysfunction, hypertrophy, and fibrosis were augmented in apelin knockout (Apelin KO) mice.

## 2. Results

### 2.1. Apelin Depletion Does Not Affect Ang II-Induced Hypertension in Aged Mice

**Figure 1:**
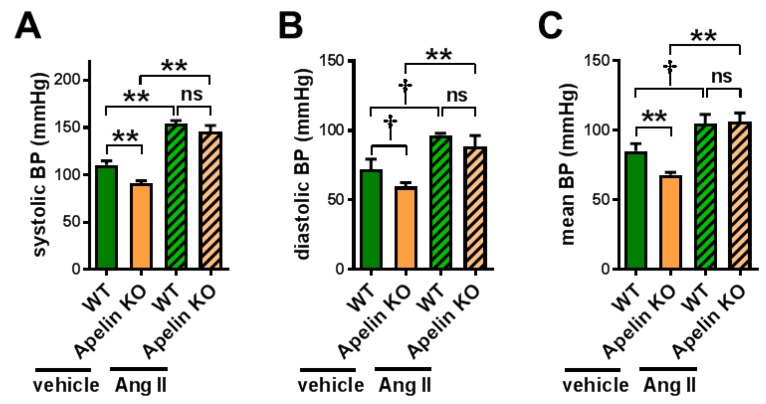
Blood pressure measurements in Ang II-treated mice. Wild type (WT) and apelin knockout (Apelin KO) mice at 12 months of age were continuously infused with either vehicle or angiotensin II (Ang II) for 2 weeks using osmotic minipumps (Ang II, 1 mg/kg/day) and measured for blood pressure using the tail-cuff method. Systolic (**A**), diastolic (**B**), and mean (**C**) blood pressures are shown. *n* = 4–6 per group. All values are mean ± SEM. † *p* < 0.1; * *p* < 0.05; ** *p* < 0.01. ns: not significant.

We first examined whether Ang II levels were affected in Apelin KO mice by subjecting the plasma to an analysis of liquid chromatography with tandem mass-spectrometry (LC-MS/MS), also called a RAS fingerprint. However, Ang II levels were not significantly changed in aged Apelin KO mice (Figure S1A,B), though there was a trend of decreasing Ang 1–7/Ang II ratios (Figure S1C,D). We thus set out to directly examine the effects of Ang II signaling in Apelin KO mice, and we treated 12-month-old Apelin KO mice and wild type (WT) mice with Ang II or vehicle for 2 weeks using osmotic minipumps. In vehicle-treated Apelin KO mice, the blood pressure was slightly but significantly decreased compared with the WT mice (Figure 1A). When the mice were continuously injected with Ang II for 2 weeks, both WT and Apelin KO mice showed elevated blood pressure in a comparable manner, and there was no difference in hypertension between the mice (Figure 1A–C). Thus, endogenous apelin has little or no effects on Ang II-induced hypertension.

### 2.2. Ratio of ACE to ACE2 Was Upregulated in the Hearts of Apelin KO Mice

**Figure 2:**
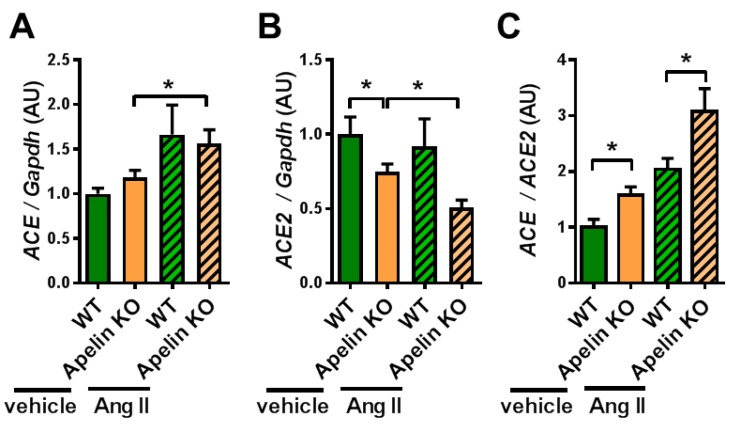
mRNA expression of ACE and ACE2 in the heart. The heart tissues harvested from Ang II-treated WT and Apelin KO mice were subjected to qRT-PCR analysis to measure mRNA expression levels. mRNA expression of ACE (**A**), ACE2 (**B**), and the ratio of ACE expression to ACE2 expression (ACE/ACE2) (**C**) are shown. *n* = 4–6 per group. All values are mean ± SEM. * *p* < 0.05.

Since the Apelin KO mice showed reduced ACE2 expression in the hearts of aged mice or mice with pressure overload stress to the left ventricle, we examined the cardiac expressions of ACE and ACE2 mRNAs. In vehicle-treated mice, ACE2 expression in the heart was downregulated by apelin depletion, whereas ACE expression was not altered (Figure 2A,B), consistent with previous results [34,36]. On the other hand, after Ang II treatment, Apelin KO mice showed a trend of decreased expression of ACE2 in the heart, but this did not reach statistical significance (Figure 2B). When we calculated the ratio of ACE to ACE2 expression, Apelin KO mice showed an increased ratio of ACE to ACE2 expression compared with WT mice after Ang II infusion as well as after vehicle treatment (Figure 2C). Thus, the loss of apelin enhances the cardiac effects of exogenous Ang II by shifting the balance of ACE and ACE2 toward the ACE dominant state.

### 2.3. Ang II Treatment Augments Heart Dysfunction in Aged Apelin KO Mice

**Figure 3:**
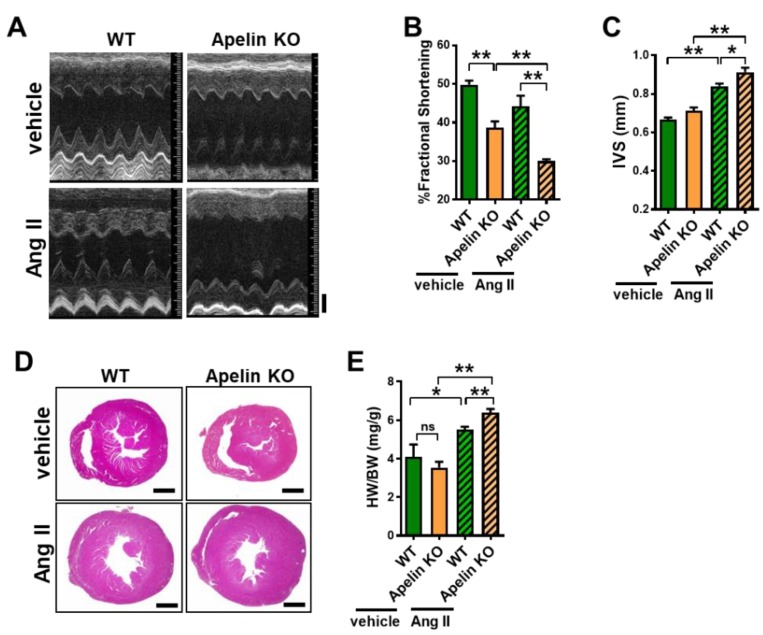
Measurements of heart function and cardiac hypertrophy in Ang II-treated mice. (**A**–**C**) Echocardiography of WT and Apelin KO mice treated with either vehicle or Ang II (*n* = 4–6 per group). Representative pictures of echocardiography (**A**), percent of fractional shortening (%FS) (**B**), and the thickness of the end-diastolic interventricular septum (IVS) (**C**) are shown. (**D**) Histology of hearts stained with hematoxylin/eosin. (**E**) Heart weight to body weight ratio (HW/BW) of WT and Apelin KO mice treated with either vehicle or Ang II (*n* = 4–6 per group). Bars indicates 1mm. All values are mean ± SEM. * *p* < 0.05; ** *p* < 0.01. ns: not significant.

When cardiac function was assessed with echocardiography, the percent of fractional shortening (%FS) was decreased in aged Apelin KO mice treated with vehicle compared to the age-matched WT control mice that received vehicle treatment (Figure 3A,B, Table 1), which was consistent with our previous results [8]. After 2 weeks of continuous Ang II infusion, WT mice showed non-significant reduction of %FS compared with vehicle-treated WT mice (Figure 3A,B, Table 1). However, Ang II-treated Apelin KO mice showed a marked reduction of %FS compared with Ang II-treated WT mice (Figure 3A,B, Table 1).

### 2.4. Loss of Apelin Exacerbates Ang II-Induced Cardiac Hypertrophy

In echocardiography, the thickness of the interventricular septum (IVS) was significantly increased in Ang II-infused Apelin KO mice (Figure 3C), suggesting that Ang II-mediated cardiac hypertrophy was enhanced in Apelin KO mice. Consistently, Apelin KO mice with Ang II infusion showed a significantly increased heart weight to body weight (HW/BW) ratio, compared with Ang II-infused WT mice (Figure 3D,E).

### 2.5. Ang II Infusion Induces Cardiac Fibrosis in Aged Apelin KO Hearts

**Figure 4:**
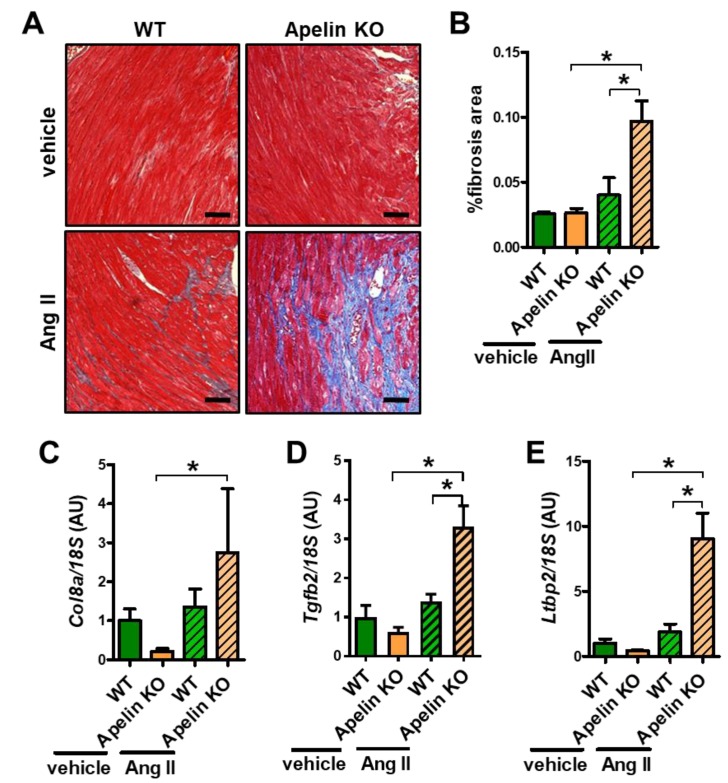
Cardiac fibrosis in Ang II-treated mice. (**A**,**B**) Histological analysis of cardiac fibrosis: Masson-trichrome staining (**A**) and quantification of %fibrosis area (**B**) in the hearts of Ang II or vehicle-treated WT and Apelin KO mice are shown (*n* = 4 per group). Bars indicates 200μm. (**C**–**E**) Real-time PCR analysis for mRNA expression of pro-fibrotic genes collagen 8a (*Col8a*) (**C**), transforming growth-factor beta 2 (*Tgfb2*) (**D**), latent transforming growth-factor beta-binding protein 2 (*Ltbp2*) (**E**), normalized with 18S respectively (*n* = 4 per group). All values are mean ± SEM. * *p* < 0.05.

We next examined cardiac fibrosis, which is the well-known outcome of enhanced Ang II signaling in the heart. Mild fibrosis of interstitial and perivascular areas were observed in Ang II-treated WT mouse hearts (Figure 4A). In contrast, Apelin KO mice with Ang II infusion exhibited massive cardiac fibrosis (Figure 4A). Quantitative analysis of fibrotic areas in the hearts showed that fibrotic areas in Ang II-treated Apelin KO mouse hearts were significantly larger than those in WT hearts with Ang II treatment (Figure 4B). Consistently, mRNA expression of the fibrosis-associated genes collagen 8a (*Col8a*), transforming growth-factor beta 2 (*Tgfb2*), and latent transforming growth-factor beta-binding protein 2 (*Ltbp2*) were significantly increased in Ang II-treated Apelin KO mice compared with WT mice (Figure 4C–E), whereas in vehicle-treated Apelin KO mice those gene expressions showed a trend to decrease, but did not reach statistical significance. Thus, Ang II augments cardiac dysfunction, hypertrophy, and fibrosis in Apelin KO mice.

### 2.6. ACE Inhibitor Improves Impaired Contractility in Aged Apelin KO Hearts

**Figure 5:**
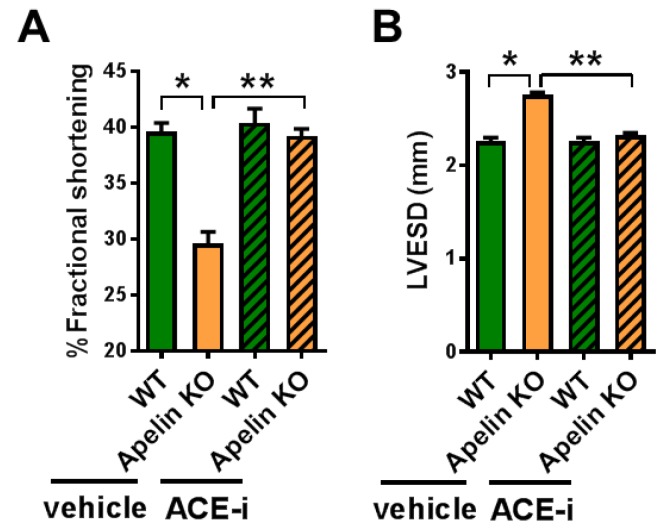
Effects of ACE inhibition on cardiac contractility in Apelin KO mice. Echocardiography of WT and Apelin KO mice treated with either vehicle or the ACE-inhibitor Captopril (ACE-i) in drinking water for 2 weeks (*n* = 5–6 per group). %fractional shortening (**A**) and diameter of end-systolic left ventricle (LVESD) (**B**) are shown. All values are mean ± SEM. * *p* < 0.05; ** *p* < 0.01.

We further examined whether Ang II depletion by ACE inhibition affects cardiac dysfunction in aged Apelin KO mice. After 2 weeks of treatment with Captopril, an ACE inhibitor (ACE-i), WT and Apelin KO mice were subjected to echocardiography. The decreased percentage of fractional shortening (%FS) and an increased end-systolic diameter of the left ventricle (LVESD) in Apelin KO mice were significantly improved by ACE-i to levels similar to those of the WT mice (Figure 5A,B; Table 2).

### 2.7. Apelin Antagonizes Ang II Effects in Primary Cardiomyocytes

**Figure 6:**
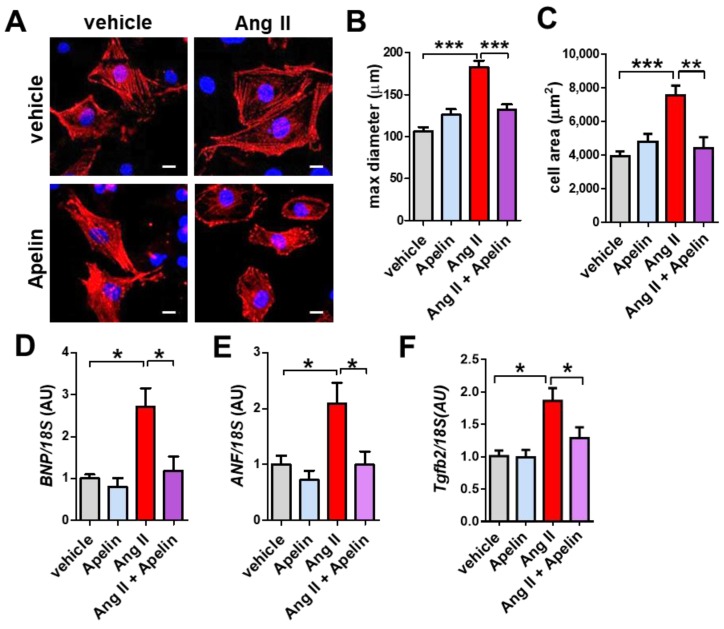
Effects of apelin and Ang II peptides in mouse cardiomyocytes. (**A**–**C**) Immunocytochemistry of primary mouse cardiomyocytes. Wild type mouse cardiomyocytes were treated with vehicle, 1 μM Ang II, 1 μM apelin, or a combination of both peptides, stained with α-actinin (red) and Hoechst (DNA, blue) and quantified. Representative pictures of cardiomyocytes (**A**), measurements of cell diameter (**B**), and cell area (**C**) are shown. Bars indicates 20μm. Values are mean ± SEM of at least 5 fields per group in 3 independent experiments. (**D**–**F**) Real-time PCR analysis for *BNP*, *ANF,* and *Tgfb2* mRNA expression in cardiomyocytes (*n* = 4 independent experiments). All values are mean ± SEM. * *p* < 0.05; ** *p* < 0.01; *** *p* < 0.001.

To determine whether Apelin antagonizes Ang II effects in vitro, we treated the isolated primary cardiomyocytes with Ang II, apelin-13, or both peptides. Ang II treatment induced hypertrophic growth of mouse cardiomyocytes, while apelin-13 did not affect the cell size (Figure 6A,B). Interestingly, combination treatment of Ang II and Apelin-13 reduced Ang II-mediated hypertrophy in cardiomyocytes (Figure 6A,B). Consistently, mRNA expression of brain natriuretic peptide (*BNP*), atrial natriuretic factor (*ANF*), and *Tgfb2* were upregulated by Ang II treatment, whereas apelin-13 significantly decreased the elevated expression of *BNP*, *ANF*, and *Tgfb2* in Ang II-treated cells to the levels of control vehicle-treated cardiomyocytes (Figure 6C–E). Thus, apelin antagonizes Ang II-mediated hypertrophy and gene expression in cardiomyocytes.

## 3. Discussion

In this study, we demonstrated that endogenous Apelin improves exogenous Ang II-induced cardiac dysfunction and pathological remodeling, as well as antagonizing endogenous Ang II-mediated impairment of heart contractility in aged mice. We also showed that apelin suppresses cellular hypertrophy and pro-fibrotic gene expression in in vitro cardiomyocytes. These data provide direct evidence that endogenous apelin is crucial to antagonize the Ang II–AT1R axis in cardiac muscle cells.

While endogenous apelin antagonizes Ang II-induced heart pathology and upregulates ACE2 expression, it is interesting to observe that Ang II-stimulated elevation of blood pressure was not further increased in Apelin KO mice but comparable to WT mice. This is a sharp contrast to the marked elevation of blood pressure in ACE2 knockout mice when treated with Ang II [37]. Although treatment with the apelin peptide antagonizes Ang II-stimulated blood pressure elevation [36], the physiological effects of endogenous apelin on the RAS seem to be biased to the heart. Consistently, our initial study of Apelin KO mice first identified the phenotype of cardiac dysfunction. In addition, ex vivo measurements of cardiac contractility showed that ACE-inhibitor treatment partly rescued the impaired contractility of Apelin KO mouse hearts (unpublished results), implicating that intrinsic cardiac RAS activation is causative of heart dysfunction. Despite numerous reports on the variety of functions of apelin, these evidences suggest that the heart is one of the major target organs for endogenous Apelin.

The pathological response during the progression of heart failure involves myocyte hypertrophy and cardiac fibrosis, which is crucially mediated by Ang II signaling. In this study, we showed that apelin inhibits Ang II-induced cellular hypertrophy and pro-fibrotic gene expression in cardiomyocytes in vitro. The results can be explained by two mechanisms: one is apelin-mediated downregulation of Ang II–AT1R signaling in the levels of receptors or intracellular signaling [35], and the other is apelin-mediated downmodulation of the ACE/ACE2 ratio and subsequent Ang II downregulation [34]. While apelin suppresses Ang II-stimulated pro-fibrotic signaling in cardiomyocytes, such as the downregulation of TGF-β expression, it was postulated that apelin directly inhibits fibrogenic responses of fibroblasts. Apelin inhibits the TGF-β-induced activation of cardiac fibroblasts and collagen production through a sphingosine kinase 1 [38]. Recently, the tissue-specific deletion of the APJ receptor in endothelial cells or myocardial cells was reported [39]. In TAC (Transverse Aortic Constriction) pressure overload models, APJ deletion in endothelial cells increased cardiac fibrosis and decreased heart contractility, whereas the cardiomyocyte-specific deletion of APJ suppressed cardiac hypertrophy and improved heart function [40]. Therefore, the favorable effects of apelin in the heart are likely mediated through complex cellular interactions and signaling. Further analysis of other cell types in the heart, such as fibroblasts, neurons, or atrial cells would be warranted for a precise understanding of apelin signaling in the heart.

Our current findings establish that endogenous apelin antagonizes the Ang II–AT1R axis in aged hearts. Apelin has been recently proposed to be an anti-aging peptide, which in mice extends the healthy life span and prevents sarcopenia, an aging-associated muscle weakness [41,42]. Currently the drugs targeting RAS, such as ACE inhibitors and angiotensin receptor blockers, are widely used in the clinic, but RAS inhibition per se does not have any inotropic affects and thus the efficacies on cardiac dysfunction in aged patients would be relatively limited. The development of therapeutic strategy to stimulate apelin-APJ signaling, which exerts positive inotrope and protective effects in the heart, would contribute to making a new class of cardiovascular medicine for aged societies.

## 4. Materials and Methods

### 4.1. Mice

Apelin knockout (apelin-/y) mice were generated as described [8] and backcrossed to C57/BL6J mice for more than 10 generations. Mice were genotyped by PCR and Southern blotting and maintained at the animal facilities of Akita University Graduate School of Medicine. All animal experiments conformed to the Guide for the Care and Use of Laboratory Animals published by the US National Institutes of Health (NIH Publication No. 85-23, revised 1996). Approval for the experiments were granted by the ethics review board of Akita University (a-1-2665, 16th March 2015).

### 4.2. Pharmacological Interventions

For Ang II treatment, 12-month-old WT and Apelin KO mice were subcutaneously infused with either vehicle or Ang II (Sigma-Aldrich, St. Louis, MI, USA) at 1 mg/kg/day for 2 weeks by osmotic minipumps (Alzet model 1002, ALZET Corp., Cupertino, CA, USA). For ACE-inhibitor treatment, 12-months-old WT and Apelin KO male mice received either vehicle or Captopril (50 mg/L) in their drinking water. Two weeks after treatment, blood pressure measurement and echocardiography were performed and the mice were sacrificed for analysis.

### 4.3. Echocardiography and Blood Pressure Measurements

Echocardiographic measurements were performed as previously described [43]. Briefly, after mice were anesthetized with isoflurane (1%)/oxygen, echocardiography was performed using Vevo770 (FUJIFILM, Tokyo, Japan) equipped with a 30-MHz linear transducer. %Fractional shortening (FS) was calculated as follows: FS = [(LVEDD − LVESD)/LVEDD] × 100. M-mode images were obtained for measurement of wall thickness and chamber dimensions with the use of the leading-edge conventions adapted by the American Society of Echocardiography. Blood pressure was measured in conscious mice by a programmable sphygmomanometer (BP-200, Softron Co. Ltd., Tokyo, Japan) using the tail cuff method after 5 days of daily training, as described previously [11].

### 4.4. LC-MS/MS Analyses for Angiotensin Peptides

Blood samples were collected with 50 units of heparin or with an optimized protease inhibitor cocktail. Plasma was kept at −80 °C until further measurement. Metabolomic profiling of Angiotensin peptides was conducted by Attoquant Diagnostics GmbH (Vienna, Austria) using technologies described previously [44]. To evaluate the activities of all RAS–peptide converting enzymes present in the plasma, we equilibrated the heparin plasma ex vivo by incubating it for 1 hour at 37 °C to obtain steady-state angiotensin peptide levels, followed by addition of a protease inhibitor cocktail (ex vivo RAS fingerprinting). Samples were then subjected to LC-MS/MS analysis using a reversed-phase analytic column (Acquity UPLC^®^ C18, Waters Co., Milford, MA, USA) operating in line with a XEVO TQ-S triple quadrupole mass spectrometer (Waters) as described [44]. For each peptide and corresponding internal standards, two different mass transitions were measured [44]. Ten angiotensin peptide metabolites could be simultaneously evaluated by this method: Ang I, Ang 1–9, Ang II, Ang III, Ang IV, Ang 1–7, Ang 1–5, Ang 2–7, Ang 3–7, and Ang 2–10.

### 4.5. Histology

Heart tissues were fixed with 4% formalin and embedded in paraffin. Five μm-thick sections were prepared and stained with hematoxylin and eosin, or Masson’s trichrome.

### 4.6. Quantitative Real-Time PCR

RNA was extracted using TRIzol reagent (Invitrogen, Carlsbad, CA, USA) and cDNA synthesized using the PrimeScript RT reagent kit (TAKARA). Sequences of the forward and reverse primers of the genes studied are shown in Supplementary Table S1. Real-time PCR was run in 96 well plates using a SYBR Premix ExTaq II (TAKARA Bio., Shiga, Japan) according to the instructions of the manufacturer. Relative gene expression levels were quantified by using the Thermal Cycler Dice Real Time System II software (TAKARA).

### 4.7. Primary Cardiomyocyte Cultures

Mouse cardiomyocytes were isolated from prenatal mouse hearts of wild type mice as described previously [34]. Briefly, prenatal mice (E17.5) were removed from pregnant mice euthanized by cervical dislocation, and prenatal mouse hearts were harvested and rapidly minced in MSS buffer (30 mM HEPES, 120 mM NaCl, 4 mM Glucose, 2 mM KCl, 1 mM KH_2_PO_4_, pH 7.6). After digestion with collagenase (Wako, Osaka, Japan) for 45 min at 35 °C, cardiomyocytes were collected, pre-plated to exclude non-cardiomyocytes, and plated on gelatinized culture dishes or plates with DMEM/F-12 (Gibco, Palo Alto, CA, USA) supplemented with 10% fetal bovine serum (Equitech Bio, Inc. Kerrville, TX, USA).

### 4.8. Statistical Analyses

Data are presented as mean values ± SEM. Statistical significance between two experimental groups was determined using Student’s two-tailed *t*-test. Comparisons of parameters among more than 3 groups were analyzed by one-way ANOVA, followed by Turkey’s post-hoc test. *p* < 0.05 was considered significant.

## Figures and Tables

**Table 1:** Echocardiography data of Apelin KO mice treated with Ang II. Values are means ± SEM. Abbreviations: (M) month; (HR) heart rate; (FS) fractional shortening; (EF) ejection fraction; (LVESD) left ventricular end-systolic diameter; (LVEDD) left ventricular end-diastolic diameter; (IVS) end-diastolic interventricular septum; (PWT) end-diastolic posterior wall thickness. * *p* < 0.05 and ** *p* < 0.01 compared with control wild type mice with vehicle. ^#^ *p* < 0.05 and ^##^ *p* < 0.01 compared to wild type mice with Ang II.

	Wild Type + Vehicle	Apelin KO + Vehicle	Wild Type + Ang II	Apelin KO + Ang II
N	4	6	4	6
age (M)	12	12	12	12
HR (bpm)	550.28 ± 24.63	550.47 ± 50.93	577.00 ± 38.03	557.67 ± 29.39
FS (%)	49.83 ± 2.05	36.33 ± 6.57 **	44.20 ± 5.52	31.35 ± 2.20 ##
EF (%)	81.30 ± 2.01	65.61 ± 9.08 **	75.46 ± 5.92	59.44 ± 3.30 ##
LVESD (mm)	2.10 ± 0.20	2.76 ± 0.36 **	2.19 ± 0.22	2.71 ± 0.31 #
LVEDD (mm)	4.18 ± 0.36	4.32 ± 0.13	3.92 ± 0.45	3.95 ± 0.38
IVS (mm)	0.67 ± 0.02	0.71 ± 0.04	0.84 ± 0.03 **	0.91 ± 0.05 #
PWT (mm)	0.79 ± 0.05	0.79 ± 0.04	1.00 ± 0.07 **	0.95 ± 0.04

**Table 2:** Echocardiography data of Apelin KO mice treated with an ACE inhibitor. Values are mean ± SEM. See Table 1 for abbreviations. The ACE inhibitor (ACE-i) Captopril was given in drinking water for 2 weeks before echocardiography. * *p* < 0.05 and ** *p* < 0.01 compared with control wild type mice. ^#^ *p* < 0.05 compared to vehicle treated Apelin KO mice.

	Wild Type + Vehicle	Apelin KO + Vehicle	Wild Type + ACE-i	Apelin KO + ACE-i
N	5	5	6	5
age (M)	12	12	12	12
HR (bpm)	476.20 ± 18.00	464.40 ± 11.50	480.60 ± 11.90	455.00 ± 15.90
FS (%)	39.60 ± 0.81	29.60 ± 1.08 **	40.40 ± 1.29	39.25 ± 0.06
LVESD (mm)	2.26 ± 0.04	2.76 ± 0.02 *	2.16 ± 0.04	2.33 ± 0.02 #
LVEDD (mm)	3.74 ± 0.05	3.90 ± 0.05 *	3.76 ± 0.06	3.85 ± 0.06
PWT (mm)	0.88 ± 0.02	0.72 ± 0.02 **	0.86 ± 0.02	0.83 ± 0.04 #

## Supplementary Materials

Supplementary materials can be found at http://www.mdpi.com/1422-0067/20/2/239/s1.

## Abbreviations

ACE2	Angiotensin-Converting Enzyme 2
AT1R	Angiotensin Type 1 Receptor
RAS	Renin–Angiotensin System
TGF-β	Transforming Growth-Factor β
