# Supplementary material for: Loss of Apelin Augments Angiotensin II-Induced Cardiac Dysfunction and Pathological Remodeling
Source: Int J Mol Sci. 2019 Jan 9;20(2):239. doi: 10.3390/ijms20020239 (PMC6358887; doi:10.3390/ijms20020239)
Supplement: Supplementary file 1 [file ijms-20-00239-s001.pdf]

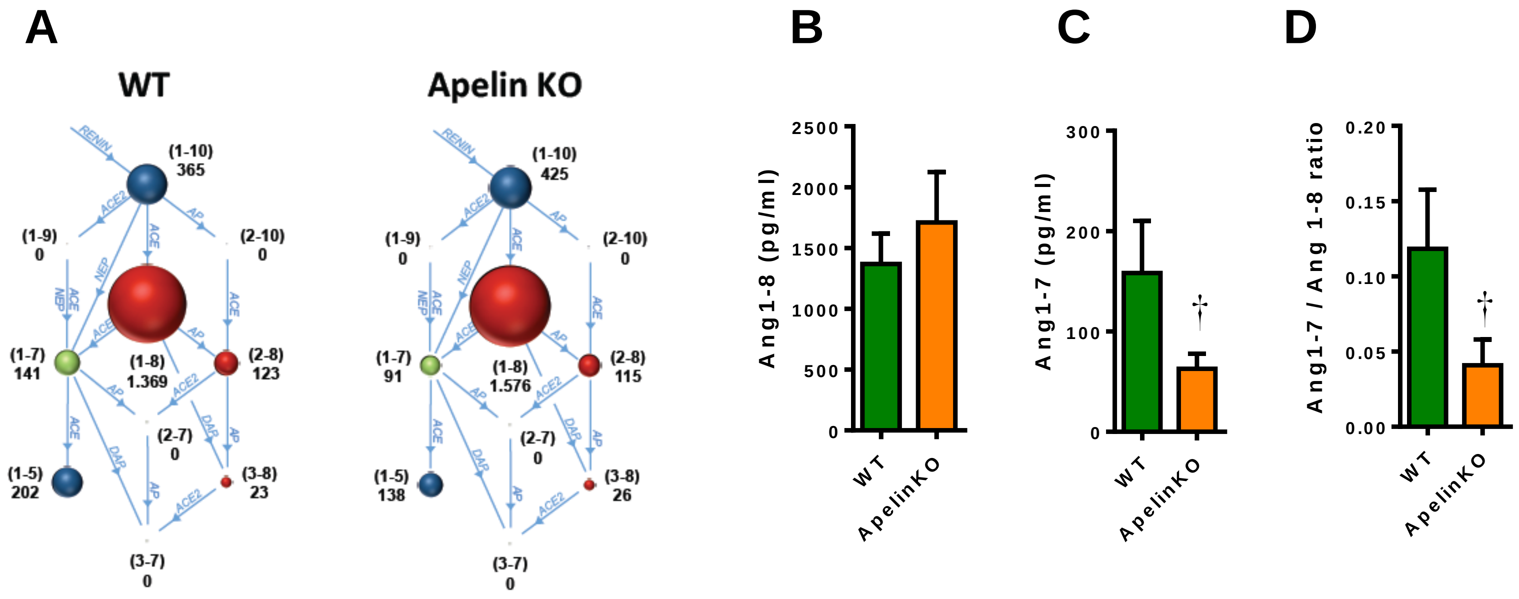

Legend

Liquid chromatography-tandem mass spectrometry (LC-MS/MS) for angiotensin metabolites in the *ex vivo* equilibrated plasma of wild type and Apelin KO mice (n = 5-8 per group). **A**, The diameter of the spheres reflects the concentration of each one of the 10 peptides: Ang (1-10), (1-9), (1-7), (1-5), (1-8), (2-7), (3-7), (2-10), (2-8), and (3-8). Values are given in picograms per milliliter next to each individual sphere; 0 pg/mL indicates concentrations below detection limits. Ang II levels (**B**), Ang 1-7 levels (C) and Ang 1-7 to Ang II ratios (**D**) are shown for the each group.

Figure S1

Supplementary Table S1

Gene	Primers	Sequence (5'-3')
BNP	Forward	ATGGATCTCCTGAAGGTGCTG
	Reverse	GTGCTGCCTTGAGACCGAA
b-MHC	Forward	GCAGCAGTTGGATGAGCGAC
	Reverse	TGCCTCCTCCAGCCTTTCAC
ANF	Forward	TGAGAAAAGCACGGAGGTATCC
	Reverse	AGAGTTTTGAAAGTTGCTCACATCA
Ace	Forward	TGAGAAAAGCACGGAGGTATCC
	Reverse	AGAGTTTTGAAAGTTGCTCACATCA
Ace2	Forward	CTACAGGCCCTTCAGCAAAG
	Reverse	TGCCCAGACCCTAGAGTTGT
Col81a	Forward	TCAGACTCATTCAGGCCGGTGC
	Reverse	CGCGCAAACTGGCTAACGGTAC
Tgfb2	Forward	AGAAGCGCGCTTTGGATGCTGC
	Reverse	TGGGACACACAGCAAGGGGAAG
Postn	Forward	TGCTCTGCTGCTGCTGTTCCTG
	Reverse	TGCTGGAGGGCACAGACGTTTG
GAPDH	Forward	CCCATCACCATCTTCCAGGA
	Reverse	GGGGCCATCCACAGTCTTCT
18S	Forward	AAACGGCTACCACATCCAAG
	Reverse	CCTCCAATGGATCCTCGTTA
